# Neurosurgery and coronavirus: impact and challenges—lessons learnt from the first wave of a global pandemic

**DOI:** 10.1007/s00701-020-04652-8

**Published:** 2020-11-21

**Authors:** Keyoumars Ashkan, Josephine Jung, Alexandra Maria Velicu, Ahmed Raslan, Mohammed Faruque, Pandurang Kulkarni, Cristina Bleil, Harutomo Hasegawa, Ahilan Kailaya-Vasan, Eleni Maratos, Gordan Grahovac, Francesco Vergani, Bassel Zebian, Sinan Barazi, Irfan Malik, David Bell, Daniel Walsh, Ranjeev Bhangoo, Christos Tolias, Sanjeev Bassi, Richard Selway, Nick Thomas, Christopher Chandler, Richard Gullan

**Affiliations:** 1 Department of Neurosurgery, King’s College Hospital, Denmark Hill, London, SE5 9RS UK; 2 Neurosciences Clinical Trials Unit, King’s College Hospital, London, UK

**Keywords:** Coronavirus, Emergency referrals, Neurosurgery, Pandemic

## Abstract

**Introduction and objectives:**

The novel severe acute respiratory syndrome coronavirus 2 (COVID-19) pandemic has had drastic effects on global healthcare with the UK amongst the countries most severely impacted. The aim of this study was to examine how COVID-19 challenged the neurosurgical delivery of care in a busy tertiary unit serving a socio-economically diverse population.

**Methods:**

A prospective single-centre cohort study including all patients referred to the acute neurosurgical service or the subspecialty multidisciplinary teams (MDT) as well as all emergency and elective admissions during COVID-19 (18th March 2020–15th May 2020) compared to pre-COVID-19 (18th of January 2020–17th March 2020). Data on demographics, diagnosis, operation, and treatment recommendation/outcome were collected and analysed.

**Results:**

Overall, there was a reduction in neurosurgical emergency referrals by 33.6% and operations by 55.6% during the course of COVID-19. There was a significant increase in the proportion of emergency operations performed during COVID-19 (75.2% of total, *n*=155) when compared to pre-COVID-19 (*n* = 198, 43.7% of total, *p* < 0.00001). In contrast to other published series, the 30-day perioperative mortality remained low (2.0%) with the majority of post-operative COVID-19-infected patients (*n* = 13) having underlying medical co-morbidities and/or suffering from post-operative complications.

**Conclusion:**

The capacity to safely treat patients requiring urgent or emergency neurosurgical care was maintained at all times. Strategies adopted to enable this included proactively approaching the referrers to maintain lines of communications, incorporating modern technology to run clinics and MDTs, restructuring patient pathways/facilities, and initiating the delivery of NHS care within private sector hospitals. Through this multi-modal approach we were able to minimize service disruptions, the complications, and mortality.

## Introduction

King’s College Hospital NHS Foundation Trust (KCH), built in 1840, is one of the largest teaching hospitals in the UK, serving a local inner-city population of 700,000 in the London districts of Southwark and Lambeth. The tertiary neurosurgical service is amongst the busiest in the country covering a regional catchment population of approximately 4 million across South East London and the county of Kent [13].

The local London boroughs have a multi-ethnic population with a comparatively high proportion of Black people (25.9%), compared to the whole London (10.9%). Amongst the largest ethnic minority groups are Black African (16.1%) and Black Caribbean (8.0%). Approximately half of the local population identifies as White British (52.2%), much lower than the national average. The socio-economic profile of the local population shows the lowest level of employment amongst all London districts [16, 21].

The novel severe acute respiratory syndrome coronavirus 2 (COVID-19) pandemic had a drastic effect on global healthcare and Europe was at the epicentre of the pandemic from March to May 2020. The UK had one of the highest death tolls across Europe and London was the worst affected. KCH had at its peak 517 inpatients with COVID-19 of which 96 were being cared for in the intensive treatment unit (ITU).

The GlobalSurg group recently published that surgical services, both elective and emergency, were severely impacted by COVID-19 and that there was an increased 30-day mortality of up to 23.8% in patients infected with COVID-19 [2]. The aim of the paper here was to examine specifically the impact of the COVID-19 pandemic on the neurosurgical delivery of care in a busy unit serving a socio-economically challenging population. We also examined the patient outcomes as well as described the strategies adopted to allow safe delivery of neurosurgical services.

## Methods

### Study design

On 18th March 2020, our unit entered the acute “COVID-19” phase, demarcated by the day of the first pre-operatively suspected COVID-19 infection in a neurosurgical patient, and when neurosurgical rota and service changes were adopted. The phase officially ended on 15th May (59 days later) when our hospital, in line with the NHS directive, entered the recovery phase. During the COVID-19 period, data were collected prospectively on all patients referred to the acute neurosurgical service, patients who were admitted electively, and patients referred to subspecialty multidisciplinary teams (MDT). These data were then compared to those obtained in the immediately preceding 59-day period (18th January to 17th March), the “pre-COVID-19”, to assess the impact of the pandemic. The manuscript was written following the Strengthening the Reporting of Observational Studies in Epidemiology (STROBE) checklist [24].

### Data collection and outcome measures

Data on the acute neurosurgical referrals were obtained through an online Patient Care System (PCS) and data on referrals to the MDTs were obtained from the relevant coordinators. Emergency and elective operating lists were sourced through the software “Galaxy Operating Theatres”. Electronic patient records were accessed to capture the following: age, gender, diagnosis, type of procedure (emergency vs. elective, adult vs. paediatric, cranial vs. spinal), subspecialty (functional, neuro-oncology, trauma, neurovascular, skull base, spinal, paediatric neurosurgery), COVID-19 infection (pre-operatively vs. post-operatively), and post-operative complications. For COVID-19-infected patients, data on ethnicity, co-morbidities, perioperative complications, ITU/hospital stay, discharge destination, and mortality were also included. Additionally, referral data included type of referral (new vs. follow-up), pathology, MDT outcome, and treatment delay/recommendation.

### Rota change, guidelines, and operating theatre adjustments

Rota and service provision changes were put in place during the pandemic on 18th of March 2020. Given the rapidly evolving Public Health England [20], NHS England [15], and the Society of British Neurological Surgeons guidelines [22], a local KCH Neurosurgery working group was established to actively review the evidence and synthesize a Guidance [12] for adjustments to elective and emergency operations, theatre ventilation, and use of personal protective equipment (PPE).

**Fig. 1:**
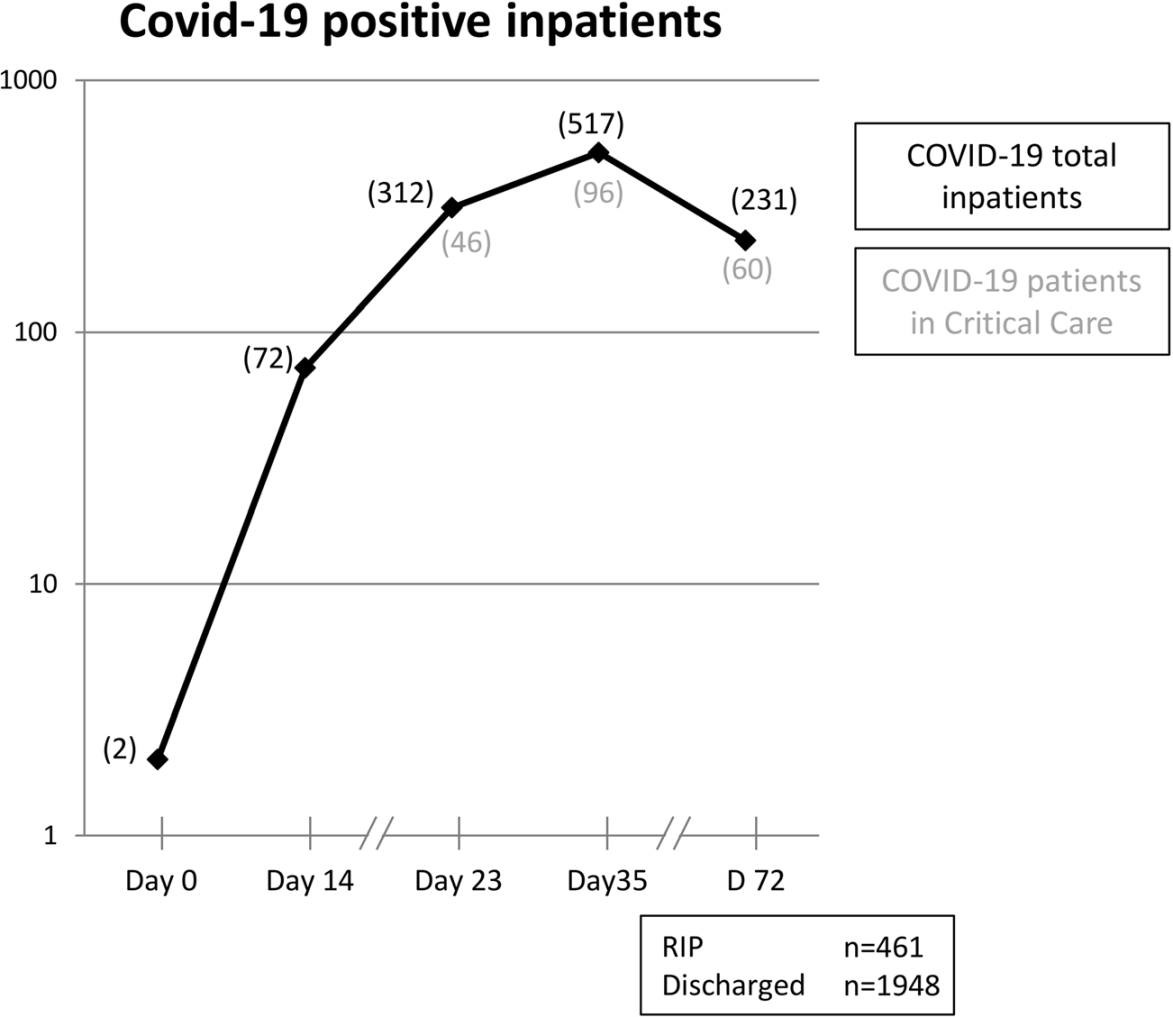
This graph describes the number of patients admitted to our hospital with COVID-19 infection. Overall number of inpatients is depicted in black, and the number of patients in intensive care is provided in grey. Day 0 was the 04th of March 2020

KCH had its first two COVID-19-positive patients on 04th of March 2020 (Fig. 1—day 0) and reached its peak on the 8th of April 2020 with 517 COVID-19-positive inpatients of which 96 were in ITU. The first change to our neurosurgical practice was put in place on 18th March 2020 where the rota was revised from a subspecialty team-based system (consisting of 1–2 neurosurgical trainees working for a specific consultant) to a twilight rota with 1 “green” team (no COVID-19 patient contact), 1 “red” team (COVID-19-positive/suspected patient contact), and 1 stand-by team in case other team members fell ill and had to consequently self-isolate. Consultant and trainee rotas both switched to a 24-h shift with one on-site consultant at all times and another on-call in reserve.

The three daily elective operating lists were condensed into one list per day from the 23rd of March 2020. Throughout the pandemic, one emergency neurosurgical theatre remained active. The allocation of theatre resources was undertaken by the Executive board of the hospital. The neurosurgical lists were arranged/triaged by the on-call neurosurgery consultant on the day. Trust guidelines recommended that ventilation in both laminar flow and conventionally ventilated theatres should remain fully switched on during surgical procedures where patients may have COVID-19 infection. All operations were performed with full PPE, including either FFP3 mask (for aerosol-generating procedures) or fluid-resistant mask (non-aerosol-generating procedures), hat, visor, gloves and fluid-resistant disposable gown. Staff training for proper donning and doffing was mandatory.

**Fig. 2:**
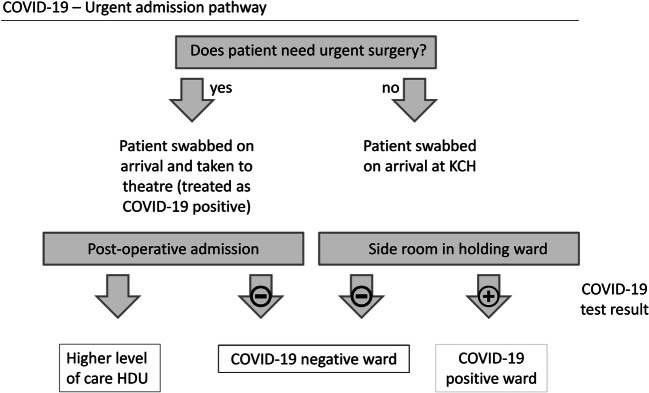
Flowchart describing admission pathway for patients requiring urgent treatment during COVID-19. All neurosurgical patients were swabbed for COVID-19 upon arrival at KCH. If urgent surgery was required, they were taken to theatre and treated as COVID-19 positive until the test result was available. If urgent surgery was not required, they were isolated in a side room in a dedicated holding ward until the COVID-19 test result was available and then either cared for in a COVID-19-positive ward or allocated to a COVID-19-negative ward based on the results

Our inpatient neurosurgical service usually consists of one purely elective 31-bedded ward, three wards for emergency and additional elective neurosurgical patients (~ 50–60 beds), and one dedicated 12-bedded neurosurgery high dependency unit (HDU). During COVID-19, out of these, one emergency admission ward and its adjacent HDU were closed to neurosurgery admissions to provide capacity for the hospital’s general COVID-19 patients. The HDU was relocated to replace one of the other neurosurgical wards with level 2 bed numbers expanded to 32. The remaining two neurosurgical wards were divided into “dirty” (COVID-19 positive) and “clean” (non-COVID-19) wards (Fig. 2).

Nearly all face-to-face outpatient appointments were cancelled and essential appointments were conducted via phone consultations. The MDT referral process remained unchanged; however, meetings took place virtually via Microsoft® Teams (Redmond, USA) from 01st April 2020.

### Statistical analysis

Descriptive statistics were used to characterize the patient population. Statistical analysis was performed using GraphPad Prism V7. Chi-squared test and the Mann-Whitney *U* test were used to assess the statistical significance of observed differences between cohorts before and during the COVID-19 pandemic.

## Results

### Emergency referrals during COVID-19

Pre-COVID-19, the median number of new acute referrals was 31 (range 17–45) per 24 h. During COVID-19, this decreased to 21 (range 10–34) per day. There was a statistically significant reduction in the overall number of referrals from 1847 to 1227 (Table 1; *p* < 0.05).

**Table 1:** Characteristics of emergency referrals and operations

Period	Pre-COVID-19	COVID-19	*p* value
Emergency referrals, total (%)	1847 (100.0)	1227 (100.0)	*p* < 0.01
Trauma/vascular	956 (51.8)	656 (53.5)	
Oncology	210 (11.4)	171 (13.9)	
Skull base	36 (1.9)	12 (1.0)	
Spinal	428 (23.2)	229 (18.7)	
Paediatrics	75 (4.1)	59 (4.8)	
Other*	141 (7.6)	100 (8.1)	
Emergency transfer, total (%)	190 (100.0)	119 (100.0)	*p* > 0.05
Neurological deficit	92 (48.4)	55 (46.2)	
GCS^a^ ≤ 8	12 (6.3)	10 (8.4)	
Age > 65 years	42 (22.1)	21 (17.6)	
Surgery, total (%)	453 (100.0)	206 (100.0)	
Emergency	198 (43.7)	155 (75.2)	*p* < 0.00001
Elective	255 (56.3)	51 (24.8)	
Cranial	263 (58.1)	134 (65.0)	ns
Spinal	190 (41.9)	72 (35.0)	
Adult	408 (90.1)	173 (84.0)	*p* < 0.05
Paediatric	45 (9.9)	33 (16.0)	
Age at operation (years)			*p* > 0.05
Mean ± SD	50 ± 21	47 ± 22	
Range	0–92	0–86	
Operative age groups (years)			*p* < 0.01
0–17	44	33	
18–65	292	178	
> 65	117	41	
COVID-19 infection			
Pre-operatively	0	4	
Post-operatively (during inpatient stay)	7	6	
Mortality, total (% of operations)	5 (1.1)	4 (2.0)	
30-day perioperative (emergencies)	5 (2.5)	3 (1.9)	
30-day perioperative (electives)	0 (0.0)	1 (2.2)	
Operations per subspecialty (adult and paediatric)			*p* < 0.01
Trauma/vascular	75 (16.6)	44 (21.4)	
Oncology	67 (14.8)	40 (19.4)	
Skull base	37 (8.2)	8 (3.9)	
Spinal	149 (32.9)	55 (26.7)	
Functional	43 (9.5)	8 (3.9)	
Other*	82 (18.1)	51 (24.8)	

*Emergency referrals and operations for other reasons such as hydrocephalus and infection were excluded from statistic calculation

^a^Glasgow coma scale

Subspecialty emergency referrals changed to proportionately fewer skull base and spinal referrals, but proportionately increased trauma, vascular, oncology, and paediatric referrals (Table 1; *p* < 0.01). Approximately 10% of patients referred as an emergency pre-COVID-19 (*n* = 190) and during COVID-19 (*n* = 119) were accepted for emergency transfer. There was no change to the definition of what constituted an emergency during or pre-COVID-19, namely being a condition that was life or limb threatening within a matter of days if left untreated. There was no significant change in the proportion of patients with neurological deficit, GCS ≤ 8, or age > 65 years being transferred to our neurosurgical centre (*p* > 0.05).

In fact, there was no statistically significant difference in age amongst patients that were admitted pre-COVID-19 (median age 53 (range 0–92) years) and during COVID-19 (median age 51 (range 0–89) years. There was however a change in gender of patients admitted pre-COVID-19 and during COVID-19 with proportionately more males being admitted during COVID-19 (59.67% compared to 52.31% pre-COVID-19, *p* < 0.05). This may potentially reflect the generally more health averse and risk-prone occupational and non-occupational behaviour amongst men resulting in acute admissions. The distribution of ethnic minority patients admitted pre-COVID-19 (8.15% Black, 3.94% Asian, 0.41% Hispanic) and during COVID-19 (9.54% Black, 3.54% Asian, 0.54% Hispanic) remained stable, albeit in a higher proportion of patients the ethnicity was not recorded during COVID-19 (24.73% pre-COVID-19, 36.24% during COVID-19), possibly reflecting the limited availability of hospital administrative support staff to record these during COVID-19.

### Emergency and elective neurosurgical operations performed before and during COVID-19

The total number of operations decreased from *n* = 453 (pre-COVID-19) to *n* = 206 (Table 1; *p* < 0.0001) with the daily median number of operations decreasing from 8 to 3. A higher percentage of emergency operations was performed during COVID-19 (75.2% of total, *n* = 155) compared to pre-COVID-19 (*n* = 198, 43.7% of total, *p* < 0.00001). There was no significant change in the proportion of cranial versus spinal operations (Table 1). Overall, significantly fewer patients aged > 65 years underwent an operation during COVID-19 (*p* < 0.01). The operations for adults and paediatrics per neurosurgical subspecialty changed significantly (*p* < 0.01) with subspecialties with a high proportion of elective work, such as functional, skull base, and spinal neurosurgery, being affected the most.

Tables 2 and 3 summarize the data on patients undergoing neurosurgery in adult and paediatric cases, respectively. The total number of adult operations performed dropped from *n* = 408 to *n* = 173 during COVID-19, with a significant amount of functional and degenerative spinal neurosurgical work being deferred or cancelled (*p* < 0.01; Table 2). The number of operations amongst the emergency subspecialties, such as trauma and vascular neurosurgery, also decreased during COVID-19 by approximately 50%; however, the case mix remained similar. The most common traumatic pathologies requiring emergency operation were chronic subdural hematoma (pre-COVID-19 *n* = 26, COVID-19 *n* = 19), vertebral fracture (pre-COVID-19 *n* = 11, COVID-19 *n* = 5), and acute subdural hematoma (pre-COVID-19 *n* = 9, COVID-19 *n* = 3). Although all the numbers decreased, the smallest drop in cases was amongst surgeries for chronic subdural haematomas, possibly related to their relatively more chronic presentation. Similarly, vascular operations decreased with fewer aneurysms being clipped during COVID-19 (*n* = 1) compared to pre-COVID-19 (*n* = 9).

**Table 2:** Number and composition of adult operations performed by subspecialty

Adult	Pre-COVID-19	COVID-19	*p* value
Number of operations in *N* (% of total)	408 (100.0)	173 (100.0)	
Functional	37 (9.1)	8 (4.6)	*p* < 0.01
DBS^a^ for Parkinson’s disease/tremor	8 (2.0)	0 (0.0)	
Intractable epilepsy/VNS^b^	18 (4.4)	1 (0.6)	
Peripheral nerve	3 (0.7)	1 (0.6)	
Baclofen pump/other	2 (0.4)	1 (0.6)	
DBS battery change	5 (2.7)	5 (2.9)	
Spinal	145 (35.5)	51 (29.5)	*p* < 0.01
Myelopathy	28 ( 6.9)	8 (4.6)	
Radiculopathy	73 (17.9)	13 (7.5)	
Cauda equina syndrome	26 (6.4)	14 (8.1)	
MSCC^d^ and spinal tumour	14 (3.4)	15 (8.7)	
Spinal haematoma and other	4 (1.0)	1 (0.6)	
Trauma	50 (12.3)	31 (17.9)	*p* > 0.05
Acute subdural hematoma	9 (2.2)	3 (1.7)	
Chronic subdural hematoma	26 (6.4)	19 (11.0)	
Extradural hematoma	4 (1.0)	2 (1.2)	
Traumatic brain injury/other	0 (0.0)	1 (0.6)	
Traumatic vertebral fracture	11 (2.7)	5 (2.9)	
Vascular	22 (5.4)	9 (5.2)	*p* > 0.05
Aneurysm	9 (2.2)	1 (0.6)	
Intracranial haemorrhage	5 (1.2)	5 (2.9)	
Ischemic stroke	1 (0.2)	1 (0.6)	
Arteriovenous malformation	7 (1.7)	0 (0.0)	
Arteriovenous fistula	1 (0.2)	2 (1.2)	
Oncology	60 (14.7)	31 (17.9)	*p* > 0.05
Low-grade glioma	4 (1.0)	1 (0.6)	
High-grade glioma	31 (7.6)	12 (6.9)	
Cerebral metastasis	6 (1.5)	6 (3.5)	
Meningioma	13 (3.2)	7 (4.0)	
Other	6 (1.5)	5 (2.9)	
Skull base	36 (8.8)	7 (4.0)	*p* > 0.05
Pituitary adenoma/apoplexy	16 (3.9)	3 (1.7)	
Sphenoid wing meningioma	5 (1.2)	2 (1.2)	
Vestibular schwannoma	6 (1.2)	0 (0.0)	
Chiari malformation	5 (1.2)	0 (0.0)	
Chondrosarcoma	2 (0.5)	0 (0.0)	
Trigeminal neuralgia	1 (0.2)	0 (0.0)	
Craniopharyngioma	1 (0.2)	2 (1.2)	
Other	58 (14.2)	37 (21.4)	*p* > 0.05
Hydrocephalus	30 (7.4)	18 (10.4)	
Primary infections	7 (1.7)	4 (2.3)	
Secondary infections	15 (3.7)	11 (6.4)	
Post-operative hematoma	2 (0.5)	0 (0.0)	
CSF leak/pseudomeningocele	4 (1.0)	4 (2.3)	

^a^Deep brain stimulation

^b^Vagal nerve stimulator

^c^Metastatic spinal cord compression

^d^Cerebrospinal fluid

**Table 3:** Number and composition of paediatric operations performed by subspecialty

Paediatric	Pre-COVID-19	COVID-19	*p* value
Number of operations in *N* (% of total)	45 (100.0)	33 (100.0)	
Functional (intractable epilepsy)	6 (13.3)	0 (0.0)	
Spinal	4 (8.9)	4 (12.1)	*p* > 0.05
Myelomeningocele	3 (6.7)	4 (12.1)	
Tethered cord	1 (2.2)	0 (0.0)	
Trauma	2 (4.4)	5 (15.2)	*p* > 0.05
Acute subdural hematoma	0 (0.0)	1 (3.0)	
Extradural hematoma	1 (2.2)	0 (0.0)	
Traumatic brain injury/intracranial haemorrhage	0 (0.0)	3 (9.1)	
Traumatic vertebral fracture	1 (1.1)	1 (3.0)	
Vascular (cavernoma)	1 (2.2)	0 (0.0)	
Oncology	7 (15.6)	9 (27.3)	*p* > 0.05
LGG	1 (2.2)	4 (12.1)	
HGG	5 (11.1)	2 (6.1)	
Medulloblastoma	1 (2.2)	2 (6.1)	
Ependymoma	0 (0.0)	1 (3.0)	
Skull base (chiari malformation)	1 (2.2)	1 (3.0)	
Other	24 (53.3)	14 (42.4)	*p* > 0.05
Hydrocephalus	20 (44.4)	14 (42.4)	
Primary infections	2 (4.4)	0 (0.0)	
Secondary infections	2 (4.4)	0 (0.0)	

Within the neuro-oncology service, the overall number of operations decreased from *n* = 60 (14.7% of total) to *n* = 31 (17.9% of total) during COVID-19. Similarly, the number of craniotomies for high-grade gliomas decreased from *n* = 31 (7.6% of total) to *n* = 12 (6.9% of total) during the pandemic.

Our skull base service was severely affected during COVID-19 with only 3 operations for pituitary adenoma/apoplexy being performed during COVID-19 (1.7% of total) from a previous number of 16 operations pre-COVID-19 (3.9% of total). No operations for trigeminal neuralgia, vestibular schwannoma, or chiari malformation were performed during COVID-19.

In functional neurosurgery, no new implantations for deep brain stimulation (DBS), spinal cord stimulation, or occipital nerve stimulation were performed. The battery change service for patients with movement disorders, however, continued albeit in the day case setting (DBS battery change *n* = 5 in both periods). All spinal surgeries decreased during COVID-19; however, notably, operations for cauda equina syndrome (pre-COVID-19 *n* = 26, COVID-19 *n* = 14) and myelopathies (pre-COVID-19 *n* = 28, COVID-19 *n* = 8) were reduced by ≥ 50% during COVID-19, whereas operations within spinal oncology category remained stable (pre-COVID-19 *n* = 14, COVID-19 *n* = 15).

### Neurovascular referral service

The total number of referrals to the vascular MDT decreased from *n* = 245 to *n* = 161 during COVID-19 (*p* < 0.05). The total number of patients referred with an intracranial aneurysm decreased from *n* = 185 (75.5% of total pre-COVID-19) to *n* = 132 (82.0% of total during COVID-19; Table 4). Within that group, referred unruptured symptomatic aneurysms remained approximately stable (~ 2.5 of total). The number of AVMs referred decreased from *n* = 34 (13.9% of total pre-COVID-19) to *n* = 8 (5.0% of total during COVID-19).

**Table 4:** Referrals to neurovascular and spinal multidisciplinary teams

Period	Pre-COVID-19	COVID-19	*p* value
Vascular referral age groups (years)			*p* < 0.01
0–17	7	5	
18–65	183	97	
> 65	55	59	
Vascular diagnosis, total (%)	245 (100.0)	161 (100.0)	*p* < 0.01
Aneurysm(s)	185 (75.5)	132 (82.0)	
Previously ruptured	79 (32.2)	22 (13.7)	
Unruptured symptomatic	6 (2.4)	4 (2.5)	
Unruptured incidental	100 (40.8)	106 (65.8)	
AVM^a^	34 (13.9)	8 (5.0)	
Previously ruptured cranial	12 (4.9)	1 (0.6)	
Unruptured cranial	20 (44.4)	7 (4.3)	
Spinal	2 (0.8)	0 (0.0)	
Cavernoma	1 (0.4)	5 (3.1)	
Other*	25 (10.2)	17 (10.6)	
Vascular treatment			
Emergency, clip/coil	6/10	1/17	*p* > 0.05
Ruptured or dissecting intracranial aneurysm	4/6	1/13	
Ruptured or symptomatic AVM^a^ or AVF^b^	2/4	0/4	
Elective, clip/coil	8/9	0/0	
Intracranial aneurysms	5/8	0/0	
AVM^a^ or AVF^b^	3/1	0/0	
Spinal MDT^c^ referrals, total (%)	526 (100.0)	248 (100.0)	*p* < 0.001
Cauda equina syndrome	21 (4.0)	26 (10.5)	
Degenerative spine	505 (96.0)	222 (89.5)	
Spinal treatment recommendation (% of total)			*p* > 0.05
Routine outpatient	334 (63.5)	151 (60.9)	
Urgent outpatient	24 (4.6)	7 (2.8)	
Conservative or other	168 (31.9)	90 (36.3)	

*Intracranial haemorrhage—no abnormality, non-aneurysmal subarachnoid haemorrhage, stenosis, family history

^a^Arteriovenous malformation

^b^Arteriovenous fistula

^c^Multidisciplinary team

Sixteen patients underwent emergency treatment pre-COVID-19, 6 of those underwent open surgery and 10 underwent endovascular treatment. During COVID-19, only 1 patient underwent emergency open surgery and 17 patients underwent emergency endovascular treatment. All elective surgery was halted during COVID-19 (Table 4).

### Neuro-oncology and skull base referral service

The total number of referrals to the neuro-oncology MDT decreased from *n* = 443 to *n* = 275 during COVID-19 (*p* < 0.05) with the median number of referrals per MDT dropping from 53 ± 11.63 to 37 ± 9.58. There was no significant change in the ratio of new to follow-up referrals during these periods. Equally, there was no significant change in the treatment recommendation provided for patients with high-grade gliomas (HGG), low-grade gliomas (LGG), and cerebral metastases (CM) (*p* > 0.05; Table 5). However, there was a significant treatment delay (surgery or adjuvant therapy), *n* = 4 patients (0.9% of total) pre-COVID-19 versus *n* = 32 patients (11.6% of total, *p* < 0.00001) during COVID-19, with patients with a meningioma affected more severely (*n* = 16 overall) compared to patients with gliomas or malignant tumours (*n* = 7 HGG, *n* = 2 LGG, *n* = 3 CM). The most common reasons for treatment delay were surgery delay due to COVID-19 because of resource limitations (*n* = 26), secondly unrelated reasons (*n* = 7), patient preference due to fear of infection (*n* = 2) and chemotherapy delay due to COVID-19 (*n* = 2).

**Table 5:** Referrals to neuro-oncology and skull base multidisciplinary teams

Period	Pre-COVID-19	COVID-19	*p* value
Neuro-oncology diagnosis, total (%)	443 (100.0)	276 (100.0)	*p* > 0.05
New referrals	298 (67.3)	185 (67.0)	
High-grade glioma	65 (14.7)	33 (12.0)	
Low-grade glioma	22 (5.0)	9 (3.3)	
Cerebral metastasis	80 (18.1)	60 (21.7)	
Meningioma	59 (13.3)	37 (13.4)	
Other*	72 (16.3)	46 (16.7)	
Follow-up (including post-operative)	145 (32.7)	91 (33.0)	
High-grade glioma	34 (7.7)	26 (9.4)	
Low-grade glioma	11 (2.5)	8 (2.9)	
Cerebral metastasis	41 (9.3)	32 (11.6)	
Meningioma	35 (7.9)	17 (6.2)	
Other*	24 (5.4)	8 (2.9)	
Treatment recommendation			
High-grade glioma, total	99	59	*p* > 0.05
Surgery, %	32 (32.3)	12 (20.3)	
Monitoring, conservative or other, %	67 (67.7)	47 (79.7)	
Low-grade glioma, total	33	17	*p* > 0.05
Surgery, %	6 (18.2)	4 (23.5)	
Monitoring, conservative or other, %	27 (81.8)	13 (76.5)	
Cerebral metastasis, total	121	92	*p* > 0.05
Intervention (surgery/SRS^a^), %	30 (7/23) (24.8)	32 (6/26) (34.8)	
Monitoring, conservative or other, %	91 (75.2)	60 (65.2)	
Skull base diagnosis, total (%)	329 (100.0)	101 (100.0)	*p* < 0.05
New referrals	112 (34.0)	48 (47.5)	
Meningioma	25 (7.6)	14 (13.9)	
Vestibular schwannoma	17 (5.2)	7 (6.9)	
Pituitary adenoma and/or apoplexy	29 (8.8)	14 (13.9)	
Chiari malformation	11 (3.3)	2 (2.0)	
Other^&^	30 (9.1)	11 (10.9)	
Follow-up (incl. post-operative)	217 (66.0)	53 (52.5)	
Meningioma	68 (20.7)	20 (19.8)	
Vestibular schwannoma	56 (17.0)	10 (9.9)	
Pituitary adenoma and/or apoplexy	51 (15.5)	17 (16.8)	
Chiari malformation	3 (0.9)	0 (0.0)	
Other^&^	39 (11.9)	6 (5.9)	
Treatment recommendation			
Meningioma, total	93	34	*p* < 0.05
Intervention (surgery/SRS^a^), %	9 (5/4) (9.7)	9 (7/2) (26.5)	
Interval imaging, %	84 (90.3)	25 (73.5)	
Vestibular schwannoma, total	73	17	*p* > 0.05
Intervention (surgery/SRS^a^), %	9 (6/3) (12.3)	2 (1/1) (11.8)	
Interval imaging, %	64 (87.7)	15 (88.2)	
Pituitary adenoma/apoplexy, total	80	31	*p* > 0.05
Surgery, %	16 (20.0)	3 (9.7)	
Interval imaging, %	64 (80.0)	28 (90.3)	

*Ependymoma, nerve sheath tumour, haemangioblastoma, arachnoid/colloid cyst, etc.

^a^Stereotactic radiosurgery

^&^Chondrosarcoma, chordoma, craniopharyngioma, etc.

Within the skull base service, the number of referrals was significantly reduced from *n* = 329 to *n* = 101 during COVID-19 (*p* < 0.001). Notably, the overall number of patients referred for a pituitary adenoma reduced from *n* = 80 to *n* = 31 (*p* < 0.001). Out of those, *n* = 11 were referred with pituitary apoplexy pre-COVID-19 and *n* = 3 during COVID-19. There was no statistically significant difference in treatment recommendation between patients referred pre-COVID-19 and during COVID-19 for patients with vestibular schwannoma and pituitary adenoma/apoplexy. However, in a higher proportion of patients referred with a meningioma during COVID-19, active treatments such as surgery or SRS, instead of monitoring were recommended, possibly indicating that larger or more clinically symptomatic lesions were being referred during the COVID-19 period (*p* < 0.05; Table 5). All meningioma cases, where specialist intervention was recommended, were located in the medial sphenoid wing. Surgery was recommended to 3 patients with pituitary adenomas during COVID-19: 1 had pituitary apoplexy, 1 had progressively deteriorating vision, and in 1 patient, the pituitary mass had progressed over a short period of time and turned out to be a metastasis. Surgical intervention was deferred in *n* = 5 for sphenoid wing meningiomas, and *n* = 15 for pituitary adenoma.

### Referrals to the spinal MDT

The total number of referrals to the spinal MDT decreased significantly during COVID-19 from *n* = 526 to *n* = 248 (*p* < 0.001; Table 4). The proportion of patients referred with cauda equina syndrome increased significantly from 4.0% (*n* = 21) to 10.5% (*n* = 26, *p* < 0.001). There was no statistically significant difference between spinal MDT treatment recommendations before and during COVID-19 (Table 4).

### Functional and paediatric neurosurgery

The number of functional neurosurgery MDTs was reduced from twice-weekly pre-COVID-19 to 3 during COVID-19. No elective functional neurosurgery took place during COVID-19 although battery replacement for movement disorder patients continued (Table 2).

In the paediatric service, the total number of operations performed was not as severely affected as the adult service (pre-COVID-19 *n* = 45, during COVID-19 *n* = 33) but the case load amongst the subspecialties changed (Table 3). In particular, trauma cases increased from *n* = 2 (4.4% of total pre-COVID-19) to *n* = 5 (15.2% of total during COVID-19). Oncology operations also increased from *n* = 7 (15.6% of total) to *n* = 9 (27.3% of total) during COVID-19. No functional or neurovascular operations were performed during COVID-19 within our paediatric cohort.

### Surgical outcomes and COVID-19 infections in neurosurgical patients

Overall, 30-day perioperative mortality remained low during COVID-19 (*n* = 4, 2.0%) compared to pre-COVID-19 (*n* = 5, 1.1%; Table 1). Within emergency operations, 30-day perioperative mortality was lower during COVID-19 (1.9%, *n* = 3) compared to pre-COVID-19 (2.5%, *n* = 5), partly reflecting the process of patient selection with higher threshold for transfer and surgery in critically ill patients during the COVID-19 period. The single elective mortality in the COVID-19 period related to a 28-year-old patient with a solitary CM who subsequently passed away due to leptomeningeal disease (Table 1; 30-day perioperative elective mortality during COVID-19 *n* = 1, 2.2%).

There were 17 neurosurgical patients who were diagnosed with COVID-19 either pre-operatively (*n* = 4) or post-operatively (operation pre-COVID-19 *n* = 7, operation during COVID-19 *n* = 6; all patients tested negative before surgery; Table 1), representing 2.6% of total neurosurgical operations. Out of these 17 patients, 6 (35.3%) were from a black and minority ethnic (BAME) background (Table 6) and one of these BAME patients died of post-operative COVID-19 infection (accounting for 20.0% of all deaths after emergency operation). This was an 86-year-old Asian man with hypertension who underwent burr hole drainage of a chronic subdural hematoma but developed COVID-19 infection 15 days post-operatively and died 6 days later of COVID-19-related pneumonia. This was the only single mortality of a neurosurgical patient with COVID-19 infection within our cohort. There was no difference in the ethnic mix of our patients between the pre-COVID and COVID periods. The median age amongst these 17 patients was 63 ± 15.44 years and male:female ratio was 10:7. Overall, *n* = 4 patients (23.5%) were admitted to ITU because of COVID-19-related complications. The majority of patients who were infected with COVID-19 had underlying co-morbidities such as hypertension and diabetes mellitus, and all patients admitted to ITU had underlying health problems. Out of the 13 patients who developed post-operative COVID-19 infection, 53.8% (*n* = 7) had suffered from a post-operative complication (*n* = 6 wound infection, *n* = 1 hematoma, *n* = 1 CSF leak) with a median time to post-operative infection of 18 ± 9.5 days. The median length of stay for the 17 patients diagnosed with COVID-19 was 36 ± 23.97 days; 4 (23.5%) were discharged to a rehabilitation unit, and 11 (64.7%) were discharged home.

**Table 6:** Characteristics of neurosurgical patients with COVID-19 infection

Nr.	Age, sex	Ethnic group	Admission		Co-morbidities	Diagnosis	Procedure	Complications	ITU	Outcome	LoS
			Type	COVID							
1	54, M	White	Em	d0	HTN	Pineal lesion	Endoscopic third ventriculostomy + biopsy	None	Yes	Home	46 days
2	38, M	Black	Em	d0	Illicit drugs	Subarachnoid haemorrhage	External ventricular drain	None	Yes	Home	15 days
3	54, M	White	Em	d0	Pancytopaenia	Colloid cyst	Endoscopic resection	None	No	Home	27 days
4	41, M	White	Em	d0	HTN, NIDDM, asthma	Intracranial haemorrhage	Craniotomy	None	Yes	Home	55 days
5	59, M	White	Elec	d16	Metastatic cancer	Cerebral metastasis	Craniotomy	None	No	Home	24 days
6	67, M	White	Em	d34	HTN, NIDDM, Cholesterol↑	Meningitis	External ventricular drain	None	Yes	Rehab	90 days
7	51, M	White	Em	d20	None	Degenerative spine	Lumbar fixation	Cerebrospinal fluid leak, wound infection	No	Home	36 days
8	72, M	White	Elec	d31	None	Parkinson’s	Deep brain stimulation	Wound infection	No	Inpatient	94 days
9	72, F	Asian	Elec	d42	HTN, NIDDM	Meningioma	Craniotomy	Wound infection	No	Home	65 days
10	86, M	Asian	Em	d15	HTN, dementia	Subdural hematoma	Burr hole evacuation	None	No	RIP	21 days
11	64, F	White	Em	d14	None	Meningioma	Craniotomy/cranioplasty	Wound infection	No	Rehab	29 days
12	86, F	White	Em	d18	HTN, AF, cholesterol↑	Spinal hematoma	Decompression	Wound infection	Yes	Rehab	31 days
13	48, F	Black	Elec	d21	Asthma, cholesterol↑	Meningioma	Craniotomy	Haematoma	Yes	Rehab	67 days
14	66, M	White	Em	d14	Metastatic cancer	Metastatic cord compression	Decompression + fixation	None	No	Home	22 days
15	33, F	Hispanic	Elec	d11	None	High-grade glioma	Craniotomy	Wound infection	No	Home	23 days
16	81, F	Black	Em	d11	HTN, NIDDM, AF	Cauda equina syndrome	Laminectomy + discectomy	None	No	Home	37 days
17	63, F	White	Em	d24	None	Meningitis	Endoscopic third ventriculostomy + Rickham	None	No	Home	47 days

Admission: Type—Em(ergency) vs. Elec(tive); COVID—time to COVID-19 infection from day of admission (days)

*ITU*, admission to intensive treatment unit; *LoS*, length of inpatient hospital stay (days)

Co-morbidities: hypertension (HTN), non-insulin-dependent diabetes mellitus (NIDDM), atrial fibrillation (AF)

## Discussion

### Impact of COVID-19 on neurosurgical referrals and service

Overall, we saw a reduction in acute referrals during COVID-19 by approximately 33.6% and in the number of operations performed by approximately 55.6%. This is comparable to the published literature where a reduction of more than 50% has been described by 226 respondents from more than 60 countries [9]. Mathiesen et al. demonstrated in a European snapshot that in 80% of respondents (20 neurosurgical departments), neurosurgical beds and neuro-intensive care beds were rationalized by postponing elective surgery, fewer acute traumatic brain injuries and subarachnoid haemorrhages admissions, and changing surgical indications in order to ration resources [14]. Although we did not see a statistically significant difference in patients with neurological deficit, GCS ≤ 8, or age > 65 years being transferred to our neurosurgical unit during COVID-19, there was a trend towards admitting fewer elderly patients with depressed GCS. This may represent the tendency to protect the ventilated ITU bed capacity by limiting the admission of patients with extremely poor prognosis. Additionally, referrals to our neurosurgical subspecialist MDTs were decreased although subspecialties with a more elective case mix (skull base, spine, functional) were worse affected than those with a more urgent case mix (neuro-oncology, neurovascular, paediatrics).

Hecht et al. described a similar percentage reduction for neurosurgical emergency admissions although across all subspecialties (*p* = 0.0007) during COVID-19 [6]. In keeping with their findings, we observed a reduction in the total numbers of patients presenting with sub-/epidural hematomas, traumatic vertebral fractures, and hydrocephalus (as described in Table 2). Interestingly, operations for cauda equina syndrome and spinal myelopathy decreased during COVID-19, which could be either due to a delay in presentation to the emergency department (patient-related factors) or due to a delay in referral through the general practitioner as many had their practice disrupted during COVID-19 (physician-related factors). In contrast though, we observed a similar number of patients with neurovascular emergencies (i.e. ruptured intracranial aneurysm) presenting during and pre-COVID-19 with a higher proportion undergoing endovascular treatment during COVID-19; the treatment decision was not based on resource allocation but was merely a result of the type of aneurysms presenting during the COVID-19 period. However, this also helped to reduce theatre usage and length of hospital stay.

Although there was a reduction in the referrals to our subspecialist MDTs, we developed strategies to avoid critical delays. For example, in neuro-oncology, we actively reached out to our referring centres to encourage continued referral of patients. Importantly, there was no significant change in treatment recommendation with regard to gliomas and CMs in our cohort (*p* > 0.05; Table 5). In terms of delivery of surgery, we mitigated the effects of reduced theatre space during COVID-19 in our unit by securing additional theatre capacity in the private healthcare sector, contracted through the NHS. This also meant that the operations performed during COVID-19 in the private sector (*n* = 26) were provided by a “clean” neurosurgical team to a “clean” (COVID-19 negative) cohort of neuro-oncology patients, further reducing the risks. Similar arrangements were used to maintain the delivery of the degenerative spine disease for medically refractory neural compression, with no significant difference in waiting times between pre-COVID-19 and COVID-19 periods, matching the capacity to referrals (*p* > 0.05, median waiting times 28 and 27 weeks respectively).

### Impact of COVID-19 on subspecialties and neurosurgical training

There has been little focus on the impact of COVID-19 on neurosurgical training [1]. During the COVID-19, and as part of restructuring the services to release capacity to deal with COVID-19, most of our neurosurgical trainees were redeployed to ITU or COVID-19 wards. This combined with the fact that fewer operations were performed, meant a reduction in training opportunities. There was a move to teach through Zoom (Communications Technology Company, San Jose, CA) conference calls within our unit as elsewhere [19] to keep theoretical knowledge up-to-date; however, this cannot replace actual operative experience. This is further confounded as certain subspecialties have had to modify their operative techniques based on recommendations from various neurosurgical societies, particularly to avoid approaches through the respiratory tract (e.g. trans-sphenoidal surgery) or to limit the use of aerosol-generating instruments (including drills, ultrasonic aspirator) [3, 7].

As part of an effort to reduce physical contacts, in our unit, face-to-face outpatient clinic appointments were almost exclusively changed to telephone. The majority of telephone clinics were for patients being under regular follow-up with stable imaging findings and clinical course. All postponed or cancelled elective patients were equally kept under close telephone follow-up and prioritized for re-scheduling according to disease/symptom severity. Follow-up outcomes included further telephone consultations, repeat imaging, face-to-face assessment, or rescheduled surgery. None of these patients required emergency admission. Only patients who required neurosurgical intervention were seen in face-to-face clinic in order to be pre-assessed for surgery. Overall, this system worked well and may have potential implications on outpatient management for the future [4, 10]. In our experience, remote access platforms such as Attend Anywhere® (Melbourne, Australia) or secure patient online chat-rooms such as The Brain Tumour Charity’s BRIAN [23] provide invaluable tools in keeping contact with patients.

### COVID-19-positive patients and perioperative mortality

The GlobalSurg reported a 30-day perioperative mortality of 23.8% amongst COVID-19 patients undergoing emergency or elective surgery [2]. In contrast, within our cohort, 30-day perioperative mortality remained low during COVID-19 (2.0%). In fact, none of our four neurosurgical patients that underwent surgery whilst infected with COVID-19 died. We noted that 13 patients (2.0%) who underwent neurosurgery before and during COVID-19 were infected with COVID-19 whilst being an inpatient; however, no single factor could be identified to trace the cause of these post-operative inpatient infections. Importantly, the majority of these patients had underlying co-morbidities or suffered a post-operative complication, hence making them more susceptible to COVID-19 infection [5]. There is new evidence suggesting that people from a BAME background are more severely affected by COVID-19 [8, 11, 17, 18]. 35.3% of our patients with COVID-19 were from a BAME background, of which one died. More data and larger cohorts are needed to further study this aspect.

### Limitations of this study

Patient numbers were limited due to the relatively short time period observed. This was however inevitable and a reflection of the dynamic nature of the pandemic since the last day of recruitment was dictated by a national change in strategy towards the next phase to restore services. Our retrospective data collection for the pre-COVID-19 phase may also be another limitation here. We further did not analyse the impact of COVID-19 on excess/indirect neurosurgical mortality due to lack of presentation to hospitals such as the case maybe, for example, for ruptured aneurysms.

## Conclusions

There was a reduction in neurosurgical referrals by 33.6% and operations by 55.6% during the course of COVID-19. The 30-day perioperative mortality, however, remained low at 2.0%, considerably lower than that in other published series [2], with the majority of patients who contracted post-operative COVID-19 infection having underlying co-morbidities and/or suffering from post-operative complications. Despite the challenges, capacity to treat patients requiring urgent or emergency neurosurgical care was maintained at all times. The strategies we adopted allowed creation of new capacity and safe delivery of neurosurgical care, with restructuring the patient pathways and facilities into COVID-19 positive and non-COVID-19, arguably as the most important step. We strongly believe our multi-modal approach was the key to minimize the disruptions, complications, and mortality and that lessons learned will have direct relevance for neurosurgical care during the current and future pandemics.
